# Generalized Weyl–Heisenberg Algebra, Qudit Systems and Entanglement Measure of Symmetric States via Spin Coherent States

**DOI:** 10.3390/e20040292

**Published:** 2018-04-17

**Authors:** Mohammed Daoud, Maurice R. Kibler

**Affiliations:** 1 Department of Physics, Faculty of Sciences Ain Chock, University Hassan II, Casablanca 91 000, Morocco; 2 Groupe Théorie, Institut de Physique Nucléaire, CNRS/IN2P3, 69622 Villeurbanne, France; 3 Faculté des Sciences et Technologies, Université Claude Bernard Lyon 1, 69622 Villeurbanne, France; 4 IDEXLYON, Université de Lyon, 69361 Lyon, France

**Keywords:** generalized Weyl–Heisenberg algebra, qubit and qudit systems, Dicke states, Perelomov coherent states, entanglement, concurrence, perma-concurrence, Fubini–Study metric, Majorana stars, Bargmann function

## Abstract

A relation is established in the present paper between Dicke states in a *d*-dimensional space and vectors in the representation space of a generalized Weyl–Heisenberg algebra of finite dimension *d*. This provides a natural way to deal with the separable and entangled states of a system of N=d−1 symmetric qubit states. Using the decomposition property of Dicke states, it is shown that the separable states coincide with the Perelomov coherent states associated with the generalized Weyl–Heisenberg algebra considered in this paper. In the so-called Majorana scheme, the qudit (*d*-level) states are represented by *N* points on the Bloch sphere; roughly speaking, it can be said that a qudit (in a *d*-dimensional space) is describable by a *N*-qubit vector (in a *N*-dimensional space). In such a scheme, the permanent of the matrix describing the overlap between the *N* qubits makes it possible to measure the entanglement between the *N* qubits forming the qudit. This is confirmed by a Fubini–Study metric analysis. A new parameter, proportional to the permanent and called *perma-concurrence*, is introduced for characterizing the entanglement of a symmetric qudit arising from *N* qubits. For d=3 (⇔N=2), this parameter constitutes an alternative to the concurrence for two qubits. Other examples are given for d=4 and 5. A connection between Majorana stars and zeros of a Bargmmann function for qudits closes this article.

## 1. Introduction

Geometrical representations are of particular interest in various problems of quantum mechanics. For instance, the Bloch representation is widely used in the context of characterizing quantum correlations in multiqubit systems [1,2,3]. This representation is based on the idea of Majorana to visualize a *j*-spin as a set of 2j points in a sphere [4]. The Bloch sphere was used in the study of entanglement quantification and classification in multiqubit systems [5,6,7]. The investigation and the understanding of quantum correlations in multipartite quantum systems are essential in several branches of quantum information such as quantum cryptography [8], quantum teleportation [9] and quantum communication [10,11].

The separability for two-qubit states can be addressed with the concept of the Wootters concurrence [12,13]. However, for multiqubit quantum systems, the measure of quantum correlations is very challenging. Several ways to understand the main features of entangled multiqubit states were employed in the literature [14,15].

Algebraic and geometrical methods were intensively used in quantum mechanics [16,17,18,19] and continue nowadays to contribute to our understanding of entanglement properties in multipartite quantum systems (for instance, see [20,21,22,23]). In this spirit, several works were devoted to geometrical analysis of entangled multipartite states to find the best measure to quantify the amount of entanglement in a multiqubit system [5,20,24,25,26,27,28,29,30]. The classification of multipartite entangled states was investigated from several perspectives using different geometrical tools [31,32,33] to provide the appropriate way to approach the quantum correlations in multiqubit states. Among these quantum states, *j*-spin coherent states are of special interest [34]. Indeed, they are the most classical (in contrast to quantum) states and can be viewed as 2j-qubit states which are completely separable. In this sense, spin coherent states can be used to characterize the entanglement in totally symmetric multiqubit systems [35].

The multipartite quantum states, invariant under permutation symmetry, have attracted a considerable attention during the last decade. This is essentially motivated by their occurrence in the context of multipartite entanglement [7,27,36,37,38,39,40,41] and quantum tomography [42,43,44]. In fact, the dimension $2^{N}$ of the Hilbert space for an ensemble of *N* qubits system reduces to N+1 when the whole system possesses the exchange symmetry. The appropriate representations to deal with the totally symmetric states are the Dicke basis [45,46,47] and Majorana representation [4].

In the present paper, we consider a realization of the generalized Weyl–Heisenberg algebra, introduced in [48,49,50], by means of an ensemble of two-qubit operators. We investigate the correspondence between the vectors of the representation space of the Weyl–Heisenberg algebra and the Dicke states. Using the decomposition properties of Dicke states, we show that the separable states are necessarily the Perelomov coherent states associated with the generalized Weyl–Heisenberg algebra. The coherent states are written as tensor products of single qubit coherent states. We also discuss the separability in terms of the permanent of the matrix of the overlap between spin coherent states.

## 2. Qubits and Generalized Weyl–Heisenberg Algebra

### 2.1. Bosonic and Fermionic Algebras

The study of bosonic and fermionic many particle states is simplified by considering the algebraic structures of the corresponding raising and lowering operators. On the one hand, for bosons, the creation operators $b_{i}^{+}$ and the annihilation operators $b_{i}^{-}$ satisfy the commutation relations

(1) $$\begin{array}{c} \left[b_{i}^{-},b_{j}^{+}\right]=\delta_{ij}I,\;\left[b_{i}^{-},b_{j}^{-}\right]=\left[b_{i}^{+},b_{j}^{+}\right]=0, \end{array}$$

where I stands for the identity operator. On the other hand, fermions are specified by the following anti-commutation relations

(2) $$\begin{array}{c} \left\{f_{i}^{-},f_{j}^{+}\right\}=\delta_{ij}I,\;\left\{f_{i}^{+},f_{j}^{+}\right\}=\left\{f_{i}^{-},f_{j}^{-}\right\}=0 \end{array}$$

of the creation operators $f_{i}^{+}$ and the annihilation operators $f_{i}^{-}$. The properties of Fock states follow from the commutation and anti-commutation relations which impose only one particle in each state for fermions (in a two-dimensional space) and an arbitrary number of particles for bosons (in an infinite-dimensional space). Following Wu and Lidar [51], there is a crucial difference between fermions and qubits (two level systems). In fact, a qubit is a vector in a two-dimensional Hilbert space as for fermions and the Hilbert space of a multiqubit system has a tensor product structure similar to for bosons. In this respect, the raising and lowering operators commutation rules for qubits are neither specified by relations of bosonic type (1) nor of fermionic type (2).

### 2.2. Qubit Algebra

The algebraic structure relations for qubits are different from those defining Fermi and Bose operators. Indeed, denoting by |0〉 and |1〉 the states of a two-level system (qubit), the lowering ($q^{-}$), raising ($q^{+}$), and number (*K*) operators defined by

(3) $$\begin{array}{ccc} q^{-}=\left|0\rangle \langle 1\right| & \Rightarrow & q^{-}|1\rangle =|0\rangle ,\;q^{-}|0\rangle =0 \end{array}$$

(4) $$\begin{array}{ccc} q^{+}=\left|1\rangle \langle 0\right| & \Rightarrow & q^{+}|0\rangle =|1\rangle ,\;q^{+}|1\rangle =0 \end{array}$$

(5) $$\begin{array}{ccc} K=|1\rangle \langle 1| & \Rightarrow & K|1\rangle =|1\rangle ,\;K|0\rangle =0 \end{array}$$

satisfy the relations

(6) $$\begin{array}{c} {\left(q^{-}\right)}^{†}=q^{+},\;K^{†}=K,\;\left[q^{-},q^{+}\right]=I-2K,\;\left[K,q^{+}\right]=+q^{+},\;\left[K,q^{-}\right]=-q^{-} \end{array}$$

(we use $A^{†}$ to denote the adjoint of *A*). Furthermore, the creation and the annihilation operators satisfy the nilpotency conditions

(7) $$\begin{array}{c} {\left(q^{+}\right)}^{2}={\left(q^{-}\right)}^{2}=0, \end{array}$$

as in the case of fermions.

We note that the commutation relations in Equation (6) coincide with those defining the algebra introduced in [52] to provide an alternative algebraic description of qubits instead of the parafermionic formulation considered in [51]. In addition, the generalized oscillator algebra $A_{\kappa }$ introduced in [49] as a particular case of the generalized Weyl–Heisenberg algebra of bosonic type [48] provides an alternative description of qubits (in [50], the algebra $A_{\kappa }$ is also denoted as $A_{\kappa }\left(1\right)$ in view of its extension to $A_{\kappa }\left(2\right)$). In fact, Equation (6) corresponds to κ=−1.

### 2.3. Qudit Algebra

To give an algebraic description of *d*-dimensional quantum systems (d≥2), we consider a set of N=d−1 qubits. We denote as $q_{i}^{+}$, $q_{i}^{-}$, and $K_{i}$ the raising, lowering, and number operators associated with the *i*-th qubit. They satisfy relations similar to Equation (6), namely,

(8) $$\begin{array}{c} {\left(q_{i}^{-}\right)}^{†}=q_{i}^{+},\;{\left(K_{i}\right)}^{†}=K_{i},\;\left[q_{i}^{-},q_{j}^{+}\right]=\left(I-2K_{i}\right)\delta_{ij},\;\left[K_{i},q_{j}^{+}\right]=+\delta_{ij}q_{i}^{+},\;\left[K_{i},q_{j}^{-}\right]=-\delta_{ij}q_{i}^{-} \end{array}$$

and

(9) $$\begin{array}{c} \left[q_{i}^{-},q_{j}^{-}\right]=\left[q_{i}^{+},q_{j}^{+}\right]=0 \end{array}$$

for i,j=1,2,⋯,N.

Let us denote as $H_{2}$ the two-dimensional Hilbert space for a single qubit. An orthonormal basis of $H_{2}$ is given by the set

{|n〉:n=0,1}.

The multiqubit $2^{N}$-dimensional Hilbert space $H_{2^{N}}$ for the *N* qubits has the following tensor product structure

$$H_{2^{N}}=H_{2}⊗H_{2}⊗\cdots ⊗H_{2}$$

(with N≥1 factors), similar to for bosons. In other words, the set

$$\{|n_{1}n_{2}\cdots n_{N}\rangle :n_{i}=0,1\;\left(i=1,2,\cdots ,N\right)\},$$

where

$$|n_{1}n_{2}\cdots n_{N}\rangle =|n_{1}\rangle ⊗|n_{2}\rangle ⊗\cdots ⊗|n_{N}\rangle ,$$

constitutes an orthonormal basis of $H_{2^{N}}$. The Dicke states shall be defined in Section 3 as linear combinations of the states $|n_{1}n_{2}\cdots n_{N}\rangle$.

We define the collective lowering, raising and number operators in the Hilbert space $H_{2^{N}}$ as follows

(10) $$\begin{array}{c} q^{-}=\sum_{i=1}^{N}q_{i}^{-},\;q^{+}=\sum_{i=1}^{N}q_{i}^{+},\;K=\sum_{i=1}^{N}K_{i} \end{array}$$

in terms of the annihilation, creation, and number operators $q_{i}^{-}$, $q_{i}^{+}$, and $K_{i}$, respectively. In Equation (10), $q_{i}^{\pm }$ should be understood as the operator $I⊗\cdots ⊗I⊗q_{i}^{\pm }⊗I⊗\cdots ⊗I$, where $q_{i}^{\pm }$ stands, among the *N* operators, at the *i*-th position from the left. It is trivial to check that

$$q^{-}|00\cdots 0\rangle =0,\;q^{+}|11\cdots 1\rangle =0.$$

The action of $q^{-}$ and $q^{+}$ on vectors $|n_{1}n_{2}\cdots n_{N}\rangle$ involving qubits |0〉 and |1〉, as for Dicke states, shall be considered in Section 3.

By using Equations (7), (9), and (10), we obtain

(11) $$\begin{array}{c} {\left(q^{-}\right)}^{k}=k!\sum_{i_{1}<i_{2}<\cdots <i_{k}}q_{i_{1}}^{-}q_{i_{2}}^{-}\cdots q_{i_{k}}^{-},\;{\left(q^{+}\right)}^{k}=k!\sum_{i_{1}<i_{2}<\cdots <i_{k}}q_{i_{1}}^{+}q_{i_{2}}^{+}\cdots q_{i_{k}}^{+} \end{array}$$

for k=1,2,⋯,N. In particular, for k=N, the relations (11) give

$${\left(q^{-}\right)}^{N}=N!q_{1}^{-}q_{2}^{-}\cdots q_{N}^{-},\;{\left(q^{+}\right)}^{N}=N!q_{1}^{+}q_{2}^{+}\cdots q_{N}^{+},$$

which lead to the nilpotency relations

(12) $$\begin{array}{c} {\left(q^{-}\right)}^{N+1}={\left(q^{+}\right)}^{N+1}=0. \end{array}$$

Equation (12) for N=1 (⇔d=2) gives back Equation (7) which is reminiscent of the Pauli exclusion principle for fermions.

In view of Equations (8) and (10), the qudit operators $q^{+}$, $q^{-}$, and *K* satisfy the commutation rules

(13) $$\begin{array}{c} \left[q^{-},q^{+}\right]=NI-2K,\;\left[K,q^{+}\right]=+q^{+},\;\left[K,q^{-}\right]=-q^{-}, \end{array}$$

which are similar to the relations defining the generalized Weyl–Heisenberg algebra $A_{\kappa }$ introduced in [49]. More precisely, let us put

(14) $$\begin{array}{c} a^{\pm }=\frac{1}{\sqrt{N}}q^{\pm }. \end{array}$$

Then, we have the relations

(15) $$\begin{array}{c} \left[a^{-},a^{+}\right]=I+2\kappa K,\;\left[K,a^{\pm }\right]=\pm a^{\pm },\;{\left(a^{-}\right)}^{†}=a^{+},\;K^{†}=K, \end{array}$$

where the parameter κ is

(16) $$\begin{array}{c} \kappa =-\frac{1}{N}. \end{array}$$

Therefore, the operators $a^{-}$, $a^{+}$, and *K* generate the algebra $A_{\kappa }$ with $\kappa =-\frac{1}{N}$. This shows that the algebra $A_{\kappa }$ can be described by a set of *N* qubits. According to the analysis in [49], since $-\frac{1}{N}<0$, the algebra $A_{\kappa }$ admits finite-dimensional representations. Indeed, we shall show that the representation constructed on the basis {|N;k〉:k=0,1,⋯,N} of the Dicke states (see Section 3) is of dimension d=N+1.

Note that the lowering and raising operators $q^{+}$ and $q^{-}$ close the following trilinear commutation relations

$$\left[q^{-},\left[q^{+},q^{-}\right]\right]=+2q^{-},\;\left[q^{+},\left[q^{+},q^{-}\right]\right]=-2q^{+}$$

similar to in a para-fermionic algebra [53]. Note that the definition in Equation (10) is also identical to the decomposition used by Green for defining para-fermions from ordinary fermions [54].

## 3. Dicke States

### 3.1. Definitions

The Hilbert space $H_{2^{N}}$ can be partitioned as

(17) $$\begin{array}{c} H_{2^{N}}=\overset{N}{\underset{k=0}{⨁}}F_{N,k}, \end{array}$$

where the sub-space $F_{N,k}$ is spanned by the orthonormal set

$$\begin{array}{c} \{|n_{1}n_{2}\cdots n_{N}\rangle :n_{1}+n_{2}+\cdots +n_{N}=k\}. \end{array}$$

Each vector $|n_{1}n_{2}\cdots n_{N}\rangle$ of $F_{N,k}$ contains N−k qubits |0〉 and *k* qubits |1〉. The dimension of the space $F_{N,k}$ is given by

$$\begin{array}{c} \dim F_{N,k}=C_{N}^{k}=\frac{N!}{k!(N-k)!}, \end{array}$$

in terms of the binomial coefficient $C_{N}^{k}$, and satisfies

$$\begin{array}{c} \dim H_{2^{N}}=\sum_{k=0}^{N}\dim F_{N,k}=2^{N}. \end{array}$$

Clearly, $F_{N,k}$ is invariant under any of the N! permutations of the *N* qubits. The orthogonal decomposition in Equation (17) of $H_{2^{N}}$ turns out to be useful in the definition of Dicke states.

To each $F_{N,k}$, it is possible to associate a Dicke state |N;k〉 which is the sum (up to a normalization factor) of the various states of $F_{N,k}$. To be more precise, let us define the Dicke state |N;k〉 as follows [46,47]

(18) $$\begin{array}{c} |N;k\rangle =\sqrt{\frac{k!(N-k)!}{N!}}\sum_{\left\{\sigma \right\}}\sigma |\underset{N-k}{\underset{︸}{00\cdots 0}}\underset{k}{\underset{︸}{11\cdots 1}}\rangle ,\;0\leq k\leq N, \end{array}$$

where the numbers 0 and 1 in the vector |00⋯011⋯1〉 are N−k and *k*, respectively. Furthermore, the summation over {σ} runs on the permutations σ of the symmetric group $S_{N}$ restricted to the identity permutation and the permutations between the 0s and 1s (the permutations between the various 0s as well as those between the various 1s are excluded, only the permutations between the 0s and 1s leading to distinct vectors are permitted). Each vector in Equation (18) involves (N−k)+k=N qubits. A Dicke state |N;k〉 is thus a normalized symmetrical superposition of the states of $F_{N,k}$. More precisely, Equation (18) means

$$\begin{array}{c} |N;k\rangle =\sqrt{\frac{k!(N-k)!}{N!}}\sum_{|x\rangle \in F_{N,k}}|x\rangle . \end{array}$$

Indeed, each Dicke state |N;k〉 and, more generally, any linear combination of the N+1 Dicke states |N;k〉 (with k=0,1,⋯,N) transform as the totally symmetric irreducible representation [N] of the group $S_{N}$ of the permutations of the N=d−1 qubits.

As a trivial example, for N=1 the Dicke states |1;0〉 and |1;1〉 are nothing but the one-qubit states |0〉 and |1〉, respectively (these qubit states are generally associated with the angular momentum states $|\frac{1}{2},\frac{1}{2})$ and $|\frac{1}{2},-\frac{1}{2})$, respectively). As a more instructive example, for N=4, we have the d=N+1=5 Dicke states

$$\begin{array}{ccc} |4;0\rangle & = & |0000\rangle ,\\ |4;1\rangle & = & \frac{1}{2}(|0001\rangle +|0010\rangle +|0100\rangle +|1000\rangle ),\\ |4;2\rangle & = & \frac{1}{\sqrt{6}}(|0011\rangle +|0101\rangle +|0110\rangle +|1001\rangle +|1010\rangle +|1100\rangle ),\\ |4;3\rangle & = & \frac{1}{2}(|0111\rangle +|1011\rangle +|1101\rangle +|1110\rangle ),\\ |4;4\rangle & = & |1111\rangle . \end{array}$$

Each vector |4;k〉 is a symmetric (with respect to $S_{4}$) linear combination of the vectors of $F_{4,k}$.

For fixed *N*, we have

$$\begin{array}{c} \langle N;k|N;\ell \rangle =\delta_{k,\ell },\;k,\ell =0,1,\cdots ,N, \end{array}$$

so that the set {|N;k〉:k=0,1,⋯,N} constitutes an orthonormal system in the space $H_{2^{N}}$. Let us denote as $G_{d}$ the space of dimension d=N+1 spanned by the N+1 symmetric vectors |N;k〉 with k=0,1,⋯,N. Then, the set {|N;k〉:k=0,1,⋯,N} is an orthonormal basis of $G_{d}$.

### 3.2. Dicke States and Representations of $A_{\kappa }$

The nilpotency relations in Equation (12) imply that the representation space of the generalized Weyl–Heisenberg algebra $A_{\kappa }$ with $\kappa =-\frac{1}{N}$, see Equations (13)–(16), is of dimension d=N+1. The representation vectors can be determined using repeated actions of the raising operator $q^{+}$ combined with the actions of the operators $q_{i}^{-}$ and $q_{i}^{+}$ defined by relations similar to Equations (3)–(5). It can be shown that these representation vectors are Dicke states. The proof is as follows.

First, the action of the operator $q^{+}$ on the ground state |00⋯0〉 of $G_{d}$ yields

$$\begin{array}{c} q^{+}|00\cdots 0\rangle =|10\cdots 0\rangle +|01\cdots 0\rangle +\cdots +|00\cdots 1\rangle \end{array}$$

or equivalently

(19) $$\begin{array}{c} q^{+}|N;0\rangle =\sqrt{N}|N;1\rangle . \end{array}$$

Second, the action of ${\left(q^{+}\right)}^{2}$ on |00⋯0〉 gives

$$\begin{array}{c} {\left(q^{+}\right)}^{2}|00\cdots 0\rangle =2\left(|110\cdots 0\rangle +|101\cdots 0\rangle +\cdots +|00\cdots 011\rangle \right) \end{array}$$

or

$$\begin{array}{c} {\left(q^{+}\right)}^{2}|N;0\rangle =\sqrt{2N(N-1)}|N;2\rangle . \end{array}$$

From repeated application of the raising operator $q^{+}$ on the state |00⋯0〉 of $G_{d}$, we obtain

(20) $$\begin{array}{c} {\left(q^{+}\right)}^{k}|N;0\rangle =\sqrt{\frac{k!N!}{(N-k)!}}|N;k\rangle . \end{array}$$

By using Equation (20), we finally get the ladder relation

(21) $$\begin{array}{c} q^{+}|N;k\rangle =\sqrt{\left(k+1)(N-k\right)}|N;k+1\rangle . \end{array}$$

Similarly, for the lowering operator $q^{-}$, we have

(22) $$\begin{array}{c} q^{-}|N;k\rangle =\sqrt{k(N-k+1)}|N;k-1\rangle . \end{array}$$

Equations (21) and (22) can be rewritten as

(23) $$\begin{array}{ccc} q^{+}|N;k\rangle & = & \sqrt{F(N,k+s+\frac{1}{2})}|N;k+1\rangle , \end{array}$$

(24) $$\begin{array}{ccc} q^{-}|N;k\rangle & = & \sqrt{F(N,k+s-\frac{1}{2})}|N;k-1\rangle , \end{array}$$

where $s=\frac{1}{2}$ and

$$\begin{array}{c} F(N,\ell )=\ell (N-\ell +1),\;0\leq \ell \leq N+1. \end{array}$$

Note that

$$\begin{array}{c} q^{+}|N;N\rangle =q^{-}|N;0\rangle =0 \end{array}$$

gives the action of the raising and lowering qubit operators on the extremal Dicke states |N;N〉 and |N;0〉 of $G_{d}$.

The Dicke states are eigenstates of the operator *K* defined in Equation (10), see also Equation (5) in the case N=1. Indeed, we have

(25) $$\begin{array}{c} K|N;k\rangle =k|N;k\rangle ,\;k=0,1,\cdots ,N, \end{array}$$

in agreement with the fact that *K* is a number operator: it counts the number of qubits of type |1〉 in the Dicke state |N;k〉. From Equations (23)–(25), we recover the commutation relations in Equation (13). Therefore, the generalized Weyl–Heisenberg algebra $A_{\kappa }$, with $\kappa =-\frac{1}{N}$, generated by the operators $\frac{1}{\sqrt{N}}q^{+}$, $\frac{1}{\sqrt{N}}q^{-}$, *K*, and I provides an algebraic description of a qudit (*d*-level) system viewed as a collection of N=d−1 qubits. In fact, the vectors of the representation space $G_{d}$ of the algebra $A_{\kappa }$ are the Dicke states |N;k〉 which are symmetric superpositions of states of a multiqubit system.

### 3.3. Decomposition of Dicke States

Let us consider again the action of $q^{+}$ on the ground state |00⋯0〉 involving *N* qubits |0〉. We have seen that

(26) $$\begin{array}{c} q^{+}|00\cdots 0\rangle =q^{+}|N;0\rangle =\sqrt{N}|N;1\rangle , \end{array}$$

see Equation (19). On another side, we have

$$q^{+}|00\cdots 0\rangle =\left(q_{1}^{+}+q_{2}^{+}+\cdots +q_{N}^{+}\right)|00\cdots 0\rangle .$$

This gives

$$q^{+}|00\cdots 0\rangle =|10\cdots 0\rangle +|01\cdots 0\rangle +\cdots +|00\cdots 1\rangle ,$$

which can be rewritten as

$$q^{+}|00\cdots 0\rangle =\left(|10\cdots 0\rangle +|01\cdots 0\rangle +\cdots +|00\cdots 1\rangle \right)⊗|0\rangle +|00\cdots 0\rangle ⊗|1\rangle ,$$

where the states |××⋯×〉 on the right-hand side member contains N−1 qubits. Thus, we get

(27) $$\begin{array}{c} q^{+}|00\cdots 0\rangle =\sqrt{N-1}|N-1;1\rangle ⊗|0\rangle +|N-1;0\rangle ⊗|1\rangle . \end{array}$$

A comparison of Equations (26) and (27) yields

(28) $$\begin{array}{c} \sqrt{N}|N;1\rangle =\sqrt{N-1}|N-1;1\rangle ⊗|0\rangle +|N-1;0\rangle ⊗|1\rangle . \end{array}$$

By applying the creation operator $q^{+}$ on both sides of Equation (28), and by using Equation (21), we obtain

$$\begin{array}{c} \sqrt{2N(N-1)}|N;2\rangle =\sqrt{2(N-1)(N-2)}|N-1;2\rangle ⊗|0\rangle +2\sqrt{N-1}|N-1;1\rangle ⊗|1\rangle . \end{array}$$

Repeating this process *k* times, we end up with

(29) $$\begin{array}{ccc} \sqrt{\frac{N!}{k!(N-k)!}}|N;k\rangle & = & \sqrt{\frac{(N-1)!}{k!(N-k-1)!}}|N-1;k\rangle ⊗|0\rangle \\ & + & \sqrt{\frac{(N-1)!}{(k-1)!(N-k)!}}|N-1;k-1\rangle ⊗|1\rangle . \end{array}$$

Equation (29) can be simplified to give

(30) $$\begin{array}{c} |N;k\rangle =\sqrt{\frac{N-k}{N}}|N-1;k\rangle ⊗|0\rangle +\sqrt{\frac{k}{N}}|N-1;k-1\rangle ⊗|1\rangle , \end{array}$$

where 0≤k≤N.

For k≠0 and k≠N, there are two terms in the decomposition of |N;k〉: one is a tensor product involving the qubit |0〉 and the other a tensor product involving the qubit |1〉. The decomposition of Equation (30) of the Dicke states |N;k〉 is trivial in the cases k=0 and k=N. For k=0 and k=N, the significance of Equation (30) is clear. These two particular cases correspond to a factorization of the Dicke state |N;k〉 for *N* qubits into the tensor product of a Dicke state for N−1 qubits with a state for one qubit.

### 3.4. Dicke States and Angular Momentum States

To close this section, a link between Dicke states and angular momentum states is in order. The Lie algebra su(2) of the group SU(2) can be realized by means of the angular momentum operators $J_{+}$, $J_{-}$, and $J_{z}$. The irreducible representation (j) of su(2) can be constructed from the set {|j,m):m=−j,−j+1,⋯,j} of angular momentum states. We know that

(31) $$\begin{array}{ccc} J_{+}\left|j,m\right) & = & \sqrt{\left(j-m)(j+m+1\right)}\left|j,m+1\right), \end{array}$$

(32) $$\begin{array}{ccc} J_{-}\left|j,m\right) & = & \sqrt{\left(j+m)(j-m+1\right)}\left|j,m-1\right), \end{array}$$

(33) $$\begin{array}{ccc} J_{z}\left|j,m\right) & = & m|j,m), \end{array}$$

according to the Condon and Shortley phase convention [55]. Let us put

$$\begin{array}{c} k=j-m,\;N-k=j+m\;\Leftrightarrow \;j=\frac{N}{2},\;m=-k+\frac{N}{2}. \end{array}$$

Therefore, the state |j,m) can be denoted as |N;k〉 since, for fixed *j*, then *N* is fixed and −j≤m≤j implies 0≤k≤N. Consequently, Equations (31)–(33) can be rewritten as

$$\begin{array}{ccc} J_{+}|N;k\rangle & = & \sqrt{k(N-k+1)}|N;k-1\rangle ,\\ J_{-}|N;k\rangle & = & \sqrt{\left(N-k)(k+1\right)}|N;k+1\rangle ,\\ J_{z}|N;k\rangle & = & \left(\frac{N}{2}-k\right)|N;k\rangle , \end{array}$$

to be compared with Equations (21), (22), and (25). This leads to the identification

$$\begin{array}{c} J_{+}=q^{-},\;J_{-}=q^{+},\;J_{z}=\frac{N}{2}I-K \end{array}$$

which establishes a link between the Weyl–Heisenberg algebra $A_{\kappa }$ with $\kappa =-\frac{1}{N}$ and the Lie algebra su(2). The rewriting of the Dicke state |N;k〉, see Equation (18), in terms of the variables *j* and *m* yields

$$\begin{array}{c} \left|j,m\right)=\sqrt{\frac{(j-m)!(j+m)!}{(2j)!}}\\ \times \sum_{\left\{\sigma \right\}}\sigma \left(\underset{j+m}{\underset{︸}{|\frac{1}{2},\frac{1}{2})⊗|\frac{1}{2},\frac{1}{2})⊗\cdots ⊗|\frac{1}{2},\frac{1}{2})}}⊗\underset{j-m}{\underset{︸}{|\frac{1}{2},-\frac{1}{2})⊗|\frac{1}{2},-\frac{1}{2})⊗\cdots ⊗|\frac{1}{2},-\frac{1}{2})}}\right), \end{array}$$

where the summation over {σ} runs on the permutations σ of the symmetric group $S_{2j}$ restricted to the identity permutation and the permutations between the states $|\frac{1}{2},\frac{1}{2})$ and $|\frac{1}{2},-\frac{1}{2})$ exclusively (only the permutations leading to distinct vectors are permitted).

## 4. Separable Qudit States

### 4.1. Factorization of a Qudit

In this section, we start from a qudit (*d*-level state) and study on which condition such a state is separable in the direct product of d−1 qubit states.

The most general state in the space $G_{d}$ can be considered as a qudit $|\psi_{d}\rangle$ constituted from N=d−1 qubits. In other words, in terms of Dicke states we have

(34) $$\begin{array}{c} |\psi_{d}\rangle =\sum_{k=0}^{N}c_{k}|N;k\rangle ,\;N=d-1,\;c_{k}\in C. \end{array}$$

We may ask the question: On which condition can the vector $|\psi_{d}\rangle$ be factorized as

$$\begin{array}{c} |\psi_{d}\rangle =|\phi_{d-1}\rangle ⊗|\varphi_{1}\rangle \end{array}$$

involving a state $|\phi_{d-1}\rangle$ for N−1 qubits and a state $|\varphi_{1}\rangle$ for one qubit?

The use of Equation (30) yields

$$\begin{array}{c} |\psi_{d}\rangle =\sum_{k=0}^{N}c_{k}\left[\sqrt{\frac{N-k}{N}}|N-1;k\rangle ⊗|0\rangle +\sqrt{\frac{k}{N}}|N-1;k-1\rangle ⊗|1\rangle \right], \end{array}$$

which can be rewritten as

$$\begin{array}{c} |\psi_{d}\rangle =|u\rangle ⊗|0\rangle +|v\rangle ⊗|1\rangle , \end{array}$$

where

(35) $$\begin{array}{c} |u\rangle =\sum_{k=0}^{N-1}c_{k}\sqrt{\frac{N-k}{N}}|N-1;k\rangle ,\;|v\rangle =\sum_{k=1}^{N}c_{k}\sqrt{\frac{k}{N}}|N-1;k-1\rangle . \end{array}$$

Clearly, the state $|\psi_{d}\rangle$ is separable if there exists *z* in C such that

(36) $$\begin{array}{c} |v\rangle =z|u\rangle . \end{array}$$

Then,

(37) $$\begin{array}{c} |\psi_{d}\rangle =|u\rangle ⊗(|0\rangle +z|1\rangle ), \end{array}$$

where

$$\begin{array}{c} \left|u\rangle \equiv \right|\phi_{d-1}\rangle ,\;|0\rangle +z|1\rangle \equiv |\varphi_{1}\rangle . \end{array}$$

It is easy to show that Equation (36) implies

$$\begin{array}{c} \sum_{k=0}^{N-1}c_{k+1}\sqrt{k+1}|N-1;k\rangle =z\sum_{k=0}^{N-1}c_{k}\sqrt{N-k}|N-1;k\rangle . \end{array}$$

Consequently, we get the recurrence relation

$$\begin{array}{c} zc_{k}\sqrt{N-k}=c_{k+1}\sqrt{k+1} \end{array}$$

that admits the solution

$$\begin{array}{c} c_{k}=c_{0}z^{k}\sqrt{C_{N}^{k}}, \end{array}$$

where the coefficient $c_{0}$ can be calculated from the normalization condition $\langle \psi_{d}|\psi_{d}\rangle =1$. This leads to

(38) $$\begin{array}{c} c_{k}=\frac{z^{k}}{{\left(1+\bar{z}z\right)}^{\frac{N}{2}}}\sqrt{\frac{N!}{k!(N-k)!}},\;k=0,1,\cdots ,N \end{array}$$

up to a phase factor. Thus, the introduction of Equation (38) into Equation (34) leads to the separable state

(39) $$\begin{array}{c} |\psi_{d}\rangle =\frac{1}{{\left(1+\bar{z}z\right)}^{\frac{N}{2}}}\sum_{k=0}^{N}z^{k}\sqrt{\frac{N!}{k!(N-k)!}}|N;k\rangle . \end{array}$$

To identify the various factors occurring in the decomposition of the separable state in Equation (39), as a tensor product, we note that the use of Equation (38) in Equation (35) gives

$$\begin{array}{c} |u\rangle =\frac{1}{\sqrt{1+\bar{z}z}}|\psi_{d-1}\rangle . \end{array}$$

Hence, Equation (37) takes the form

(40) $$\begin{array}{c} |\psi_{d}\rangle =|\psi_{d-1}\rangle ⊗|z\rangle , \end{array}$$

where

(41) $$\begin{array}{c} |z\rangle =\frac{1}{\sqrt{1+\bar{z}z}}(|0\rangle +z|1\rangle ) \end{array}$$

stands for a single qubit in the Majorana representation [4] (the vector |z〉 is nothing but a SU(2) coherent state for a spin $j=\frac{1}{2}$ as can be seen by identifying the qubits |n〉=|0〉 and |1〉 to the spin states $\left|j,m)=\right|\frac{1}{2},\frac{1}{2})$ and $|\frac{1}{2},-\frac{1}{2})$, respectively). By iteration of Equation (40), we obtain

$$\begin{array}{c} |\psi_{d}\rangle =|z\rangle ⊗|z\rangle ⊗\cdots ⊗|z\rangle , \end{array}$$

with *N* factors |z〉.

As a résumé, we have the following result. If the qudit state $|\psi_{d}\rangle$ given by Equation (34) is separable, then it can be written

(42) $$\begin{array}{c} |\psi_{d}\rangle =\frac{1}{{\left(1+\bar{z}z\right)}^{\frac{N}{2}}}\sum_{k=0}^{N}z^{k}\sqrt{\frac{N!}{k!(N-k)!}}|N;k\rangle =|z\rangle ⊗|z\rangle ⊗\cdots ⊗|z\rangle , \end{array}$$

so that $|\psi_{d}\rangle$ is completely separable into the tensor product of *N* identical SU(2) coherent states for a spin $j=\frac{1}{2}$.

### 4.2. Separable States and Coherent States

Let us consider the unitary displacement operator

$$\begin{array}{c} D\left(\xi \right)=\exp\left(\xi q_{i}^{+}-\bar{\xi }q_{i}^{-}\right),\;\xi \in C \end{array}$$

for the *i*-th qubit. The action of D(ξ) on the *i*-th qubit |0〉 can be calculated to be

(43) $$\begin{array}{c} D\left(\xi \right)|0\rangle =\cos(|\xi |)|0\rangle +\frac{\xi }{\left|\xi \right|}\sin(|\xi |)|1\rangle . \end{array}$$

By introducing

$$\begin{array}{c} z=\frac{\xi }{\left|\xi \right|}\tan\left(|\xi |\right)\;\Rightarrow \;\cos^{2}\left(|\xi |\right)=\frac{1}{1+\bar{z}z} \end{array}$$

into Equation (43), we obtain

$$\begin{array}{c} D\left(\xi \right)|0\rangle =\frac{1}{\sqrt{1+\bar{z}z}}(|0\rangle +z|1\rangle ). \end{array}$$

Hence, we have

$$\begin{array}{c} D(\xi )|0\rangle =|z\rangle , \end{array}$$

where |z〉 is the coherent state defined in Equation (41). This well-known result can be extended to the case of *N* qubits. The action of the operator $\exp(\xi q^{+}-\bar{\xi }q^{-})$, where $q^{+}$ and $q^{-}$ are given in Equation (10), on the Dicke state |N;0〉=|00⋯0〉 reads

$$\begin{array}{c} \exp(\xi q^{+}-\bar{\xi }q^{-})|N;0\rangle =|z\rangle ⊗|z\rangle ⊗\cdots ⊗|z\rangle . \end{array}$$

Therefore, the separable state $|\psi_{d}\rangle$ given by Equation (42) can be written in three different forms, namely

$$\begin{array}{c} |\psi_{d}\rangle =\frac{1}{{\left(1+\bar{z}z\right)}^{\frac{N}{2}}}\sum_{k=0}^{N}z^{k}\sqrt{\frac{N!}{k!(N-k)!}}|N;k\rangle =|z\rangle ⊗|z\rangle ⊗\cdots ⊗|z\rangle =\exp\left(\xi q^{+}-\bar{\xi }q^{-}\right)|N;0\rangle , \end{array}$$

where the last member coincides, modulo some changes of notation, with the Perelomov coherent state derived in [56] (see Formulas (122) and (123) in [56]).

## 5. Majorana Description

We now go back to the general case where the qudit state $|\psi_{d}\rangle$ of $G_{d}$ is not necessarily a separable state. This state (normalized to unity) can be written in two different forms, namely, as in Equation (34)

(44) $$\begin{array}{c} |\psi_{d}\rangle =c_{0}|N;0\rangle +c_{1}|N;1\rangle +\cdots +c_{N}|N;N\rangle ,\;N=d-1,\;\sum_{k=0}^{N}{\left|c_{k}\right|}^{2}=1 \end{array}$$

or, according to the Majorana description [4], as

(45) $$\begin{array}{c} |\psi_{d}\rangle =N_{d}\sum_{\sigma \in S_{N}}\sigma (|z_{1}\rangle ⊗|z_{2}\rangle ⊗\cdots ⊗|z_{N}\rangle ) \end{array}$$

(see Section 7 for a discussion of the equivalence between Equations (44) and (45) in the framework of the Bargmann function associated with $|\psi_{d}\rangle$ and the so-called Majorana stars). In Equation (45), the state $|z_{i}\rangle$ (with i=1,2,⋯,N) is given by Equation (41) with $z=z_{i}$. Furthermore, $N_{d}$ is a normalization factor and the sum over σ runs here over all the permutations of the symmetric group $S_{N}$. The coefficients $c_{0},c_{1},c_{2},\cdots ,c_{N}$ can be expressed in terms of the coefficients $N_{d},z_{1},z_{2},\cdots ,z_{N}$. The case where *N* is arbitrary is rather intricate. Therefore, for pedagogical reasons, we start with the case of N=2 qubits.

### 5.1. The Case N=2

For N=2 (⇔d=3), on the one hand we have

$$\begin{array}{c} |\psi_{3}\rangle =c_{0}|2;0\rangle +c_{1}|2;1\rangle +c_{2}\left|2;2\rangle ,\;\right|c_{0}{|}^{2}+|c_{1}{|}^{2}+{\left|c_{2}\right|}^{2}=1, \end{array}$$

where the Dicke states |2;k〉 with k=0,1,2 are

(46) $$\begin{array}{c} |2;0\rangle =|00\rangle ,\;|2;1\rangle =\frac{1}{\sqrt{2}}(|01\rangle +|10\rangle ),\;|2;2\rangle =|11\rangle . \end{array}$$

On the other hand,

$$\begin{array}{c} |\psi_{3}\rangle =N_{3}(|z_{1}\rangle ⊗|z_{2}\rangle +|z_{2}\rangle ⊗|z_{1}\rangle ). \end{array}$$

Therefore, we have to compare

$$\begin{array}{c} |\psi_{3}\rangle =c_{0}|00\rangle +c_{1}\frac{1}{\sqrt{2}}(|01\rangle +\left|10\rangle \right)+c_{2}|11\rangle \end{array}$$

with

$$\begin{array}{c} |\psi_{3}\rangle =N_{3}\frac{1}{\sqrt{1+|z_{1}{|}^{2}}}\frac{1}{\sqrt{1+|z_{2}{|}^{2}}}\left[2|00\rangle +\left(z_{1}+z_{2}\right)(|01\rangle +\left|10\rangle \right)+2z_{1}z_{2}|11\rangle \right]. \end{array}$$

This leads to

(47) $$\begin{array}{ccc} \frac{1}{2}c_{0} & = & N_{3}\frac{1}{\sqrt{1+|z_{1}{|}^{2}}}\frac{1}{\sqrt{1+|z_{2}{|}^{2}}},\\ \frac{1}{\sqrt{2}}c_{1} & = & N_{3}\frac{1}{\sqrt{1+|z_{1}{|}^{2}}}\frac{1}{\sqrt{1+|z_{2}{|}^{2}}}\left(z_{1}+z_{2}\right),\\ \frac{1}{2}c_{2} & = & N_{3}\frac{1}{\sqrt{1+|z_{1}{|}^{2}}}\frac{1}{\sqrt{1+|z_{2}{|}^{2}}}z_{1}z_{2}. \end{array}$$

Of course, the complex numbers $z_{1}$ and $z_{2}$ are the roots of the equation

(48) $$\begin{array}{c} z^{2}-\left(z_{1}+z_{2}\right)z+z_{1}z_{2}=0. \end{array}$$

Therefore, by combining Equations (47) and (48), we end up with the quadratic equation

(49) $$\begin{array}{c} c_{0}z^{2}-\sqrt{2}c_{1}z+c_{2}=0, \end{array}$$

so that $z_{1}$ and $z_{2}$ are given by

(50) $$\begin{array}{c} z_{1}=z_{+},\;z_{2}=z_{-},\;z_{\pm }=\frac{c_{1}\pm \sqrt{c_{1}^{2}-2c_{0}c_{2}}}{\sqrt{2}c_{0}} \end{array}$$

for $c_{0}\neq 0$ ($z=\frac{1}{\sqrt{2}}\frac{c_{2}}{c_{1}}$ for $c_{0}=0$). Observe that, when the so-called concurrence *C* defined by (see Ref. [13])

(51) $$\begin{array}{c} C=|c_{1}^{2}-2c_{0}c_{2}| \end{array}$$

vanishes, we have $z_{1}=z_{2}=z$. Therefore, the state

$$\begin{array}{c} |\psi_{3}\rangle =|z\rangle ⊗|z\rangle =\frac{1}{1+\bar{z}z}\left[|00\rangle +z(|01\rangle +\left|10\rangle \right)+z^{2}|11\rangle \right] \end{array}$$

is separable.

### 5.2. The Case *N* Arbitrary

The case *N* arbitrary is very much involved. Equations (47) and (49) for N=2 can be generalized as follows. In the general case of *N* qubits, the vector $|\psi_{d}\rangle$ of the space $G_{d}$, normalized via $\langle \psi_{d}|\psi_{d}\rangle =1$, is given by Equation (44) in terms of Dicke states or by Equation (45) in the Majorana representation. The coefficients $c_{0},c_{1},c_{2},\cdots ,c_{N}$ are connected to the complex numbers $N_{d},z_{1},z_{2},\cdots ,z_{N}$ through

$$\begin{array}{c} c_{k}=N!N_{1}N_{2}\cdots N_{N}N_{d}\sqrt{\frac{k!(N-k)!}{N!}}s_{k}\left(z_{1}z_{2}\cdots z_{N}\right), \end{array}$$

where $s_{k}\left(z_{1}z_{2}\cdots z_{N}\right)$ is the elementary symmetric polynomial (invariant under $S_{N}$) in *N* variables $z_{1},z_{2},\cdots ,z_{N}$ defined as

$$\begin{array}{c} s_{0}\left(z_{1}z_{2}\cdots z_{N}\right)=1,\;s_{k}\left(z_{1}z_{2}\cdots z_{N}\right)=\sum_{1\leq i_{1}<i_{2}<\cdots <i_{k}\leq N}z_{i_{1}}z_{i_{2}}\cdots z_{i_{k}},\;k=1,2,\cdots ,N \end{array}$$

and the normalization factors $N_{1},N_{2},\cdots ,N_{N},N_{d}$ are given by

(52) $$\begin{array}{c} N_{i}=\frac{1}{\sqrt{1+{\bar{z}}_{i}z_{i}}},\;i=1,2,\cdots ,N \end{array}$$

and

(53) $$\begin{array}{c} |N_{d}{|}^{-2}=N!\sum_{\sigma \in S_{N}}\prod_{i=1}^{N}\langle z_{i}|z_{\sigma (i)}\rangle ,\;\langle z_{i}|z_{\sigma (i)}\rangle =\frac{1+{\bar{z}}_{i}z_{\sigma (i)}}{\sqrt{\left(1+{\bar{z}}_{i}z_{i}\right)\left(1+{\bar{z}}_{\sigma (i)}z_{\sigma (i)}\right)}}. \end{array}$$

Note that

(54) $$\begin{array}{c} |N_{d}{|}^{-2}=N!\mathrm{perm}\left(A_{N}\right), \end{array}$$

where

(55) $$\begin{array}{c} \mathrm{perm}\left(A_{N}\right)=\sum_{\sigma \in S_{N}}\prod_{i=1}^{N}\langle z_{i}|z_{\sigma (i)}\rangle =\frac{1}{\prod_{j=1}^{N}\left(1+{\bar{z}}_{j}z_{j}\right)}\sum_{\sigma \in S_{N}}\prod_{i=1}^{N}\left(1+{\bar{z}}_{i}z_{\sigma (i)}\right) \end{array}$$

stands for the permanent of the N×N matrix $A_{N}$ of elements

$$\begin{array}{c} {\left(A_{N}\right)}_{ij}=\langle z_{i}|z_{j}\rangle ,\;i,j=1,2,\cdots ,N. \end{array}$$

Finally, for fixed $c_{0},c_{1},\cdots ,c_{N}$, the numbers $z_{1},z_{2},\cdots ,z_{N}$ are the roots (Majorana roots) of the polynomial equation of degree *N*

(56) $$\begin{array}{c} \sum_{k=0}^{N}{\left(-1\right)}^{k}\sqrt{\frac{N!}{k!(N-k)!}}c_{k}z^{N-k}=0, \end{array}$$

which generalizes (49).

The complete proof of Equation (56) is based on the fact that two generic qubit states $|z_{i}\rangle$ and $|z_{j}\rangle$, with j≠i, are orthogonal if and only if the variables $z_{i}$ and $z_{j}$ satisfy $z_{j}=-\frac{1}{{\bar{z}}_{i}}$. The state $|\psi_{d}\rangle$ is orthogonal to the *N* states $|-\frac{1}{{\bar{z}}_{i}}\rangle ⊗|-\frac{1}{{\bar{z}}_{i}}\rangle ⊗\cdots ⊗|-\frac{1}{{\bar{z}}_{i}}\rangle$ for i=1,2,⋯,N. This orthogonality condition shows that the variables $z_{i}$ are indeed solutions of Equation (56).

To sum up, we have the following central result. Any vector $|\psi_{d}\rangle$ in the space $G_{d}$ reads

(57) $$\begin{array}{c} |\psi_{d}\rangle =\sum_{k=0}^{N}c_{k}\left|N;k\rangle \;\Leftrightarrow \;\right|\psi_{d}\rangle =N!N_{1}N_{2}\cdots N_{N}N_{d}\sum_{k=0}^{N}\sqrt{\frac{k!(N-k)!}{N!}}s_{k}\left(z_{1}z_{2}\cdots z_{N}\right)|N;k\rangle , \end{array}$$

where the normalization factors $N_{1},N_{2},\cdots ,N_{N}$, and $N_{d}$ (with d=N+1) can be calculated from Equations (52) and (53) and the variables $z_{1},z_{2},\cdots ,z_{N}$ are given in terms of $c_{0},c_{1},\cdots ,c_{N}$ by Equation (56). Note that Equation (53) can be rewritten as

$$\begin{array}{c} |N_{d}{|}^{-2}=\frac{N!}{\left(1+{\bar{z}}_{1}z_{1}\right)\left(1+{\bar{z}}_{2}z_{2}\right)\cdots \left(1+{\bar{z}}_{N}z_{N}\right)}\sum_{\sigma \in S_{N}}\prod_{i=1}^{N}\left(1+{\bar{z}}_{i}z_{\sigma (i)}\right), \end{array}$$

so that

$$\begin{array}{c} |N!N_{1}N_{2}\cdots N_{N}N_{d}{|}^{-2}=\frac{1}{N!}\sum_{\sigma \in S_{N}}\prod_{i=1}^{N}\left(1+{\bar{z}}_{i}z_{\sigma (i)}\right). \end{array}$$

Therefore, Equation (57) becomes

(58) $$\begin{array}{c} |\psi_{d}\rangle =\sqrt{\frac{N!}{\sum_{\sigma \in S_{N}}\prod_{i=1}^{N}\left(1+{\bar{z}}_{i}z_{\sigma (i)}\right)}}\sum_{k=0}^{N}\sqrt{\frac{k!(N-k)!}{N!}}s_{k}\left(z_{1}z_{2}\cdots z_{N}\right)|N;k\rangle \end{array}$$

up to a phase factor.

As a check of the last result, note that the introduction of Equation (38) into Equation (56) yields a trivial identity. Furthermore, in the particular case where the solutions of Equation (56) are identical, i.e.,

$$\begin{array}{c} z=z_{1}=z_{2}=\cdots =z_{N}\;\Rightarrow \;s_{k}\left(zz\cdots z\right)=\frac{N!}{k!(N-k)!}z^{k}, \end{array}$$

then Equation (58) leads to the completely separable state in Equation (42). In this particular case, from Equation (55) we have

$$\begin{array}{c} \mathrm{perm}(A_{N})=N! \end{array}$$

(which is the maximum value of $\mathrm{perm}(A_{N})$). Therefore, in the general case, the quantity

(59) $$\begin{array}{c} P_{d}=\frac{1}{N!}\mathrm{perm}\left(A_{N}\right)=\frac{1}{N!}\sum_{\sigma \in S_{N}}\prod_{i=1}^{N}\langle z_{i}|z_{\sigma (i)}\rangle \end{array}$$

can be used for characterizing the degree of entanglement of the state $|\psi_{d}\rangle$.

### 5.3. The Cases d=2, 3, 4, and 5

#### 5.3.1. Case d=2

The state

$$\begin{array}{c} |\psi_{2}\rangle =c_{0}|1;0\rangle +c_{1}|1;1\rangle ,\;|1;0\rangle =|0\rangle ,\;|1;1\rangle =|1\rangle \end{array}$$

is the most general qubit (linear combination of the basic qubits |0〉 and |1〉). Of course, the notion of separability does not apply in this case.

#### 5.3.2. Case d=3

The general normalized qutrit vector is

$$\begin{array}{c} |\psi_{3}\rangle =c_{0}|2;0\rangle +c_{1}|2;1\rangle +c_{2}|2;2\rangle ,\;\sum_{k=0}^{2}{\left|c_{k}\right|}^{2}=1, \end{array}$$

where the Dicke states |2;k〉 with k=0,1,2 are

$$\begin{array}{c} |2;0\rangle =|0\rangle ⊗|0\rangle ,\;|2;1\rangle =\frac{1}{\sqrt{2}}(|0\rangle ⊗|1\rangle +|1\rangle ⊗|0\rangle ),\;|2;2\rangle =|1\rangle ⊗|1\rangle , \end{array}$$

cf. Equation (46). In the Majorana description, Equations (45), (54), and (59) give

$$\begin{array}{c} |\psi_{3}\rangle =\frac{1}{2\sqrt{P_{3}}}(|z_{1}\rangle ⊗|z_{2}\rangle +|z_{2}\rangle ⊗|z_{1}\rangle ), \end{array}$$

with

$$\begin{array}{ccc} P_{3} & = & \frac{1}{2}\mathrm{perm}\left(A_{2}\right),\\ & = & \frac{1}{2}\left(1+\right|\langle z_{1}|z_{2}\rangle {|}^{2}),\\ & = & \frac{1}{2}\frac{\left(1+{\bar{z}}_{1}z_{1}\right)\left(1+{\bar{z}}_{2}z_{2}\right)+\left(1+{\bar{z}}_{1}z_{2}\right)\left(1+{\bar{z}}_{2}z_{1}\right)}{\left(1+{\bar{z}}_{1}z_{1}\right)\left(1+{\bar{z}}_{2}z_{2}\right)}, \end{array}$$

where $z_{1}$ and $z_{2}$ are the roots of Equation (50) of the quadratic Equation (49).

It can be shown that

$$\begin{array}{c} |\langle z_{1}|z_{2}\rangle {|}^{2}=\frac{1-C}{1+C}\;\Leftrightarrow \;P_{3}=\frac{1}{1+C}\;\Leftrightarrow \;C=\frac{1}{P_{3}}-1, \end{array}$$

where the concurrence *C* for a two-qubit system is defined by Equation (51). Thus, another expression for *C* is

$$\begin{array}{c} C=\frac{\left(1+{\bar{z}}_{1}z_{1}\right)\left(1+{\bar{z}}_{2}z_{2}\right)-\left(1+{\bar{z}}_{1}z_{2}\right)\left(1+{\bar{z}}_{2}z_{1}\right)}{\left(1+{\bar{z}}_{1}z_{1}\right)\left(1+{\bar{z}}_{2}z_{2}\right)+\left(1+{\bar{z}}_{1}z_{2}\right)\left(1+{\bar{z}}_{2}z_{1}\right)}. \end{array}$$

The possible values of *C* and $P_{3}$ are

$$\begin{array}{c} \frac{1}{2}\leq P_{3}\leq 1\;\Leftrightarrow \;1\geq C\geq 0. \end{array}$$

Therefore, a vanishing concurrence C=0 (which reflects the absence of entanglement) corresponds to $P_{3}=1$; in the particular case $P_{3}=1\Leftrightarrow C=0$, we have $z=z_{1}=z_{2}\;\Leftrightarrow \;\langle z_{1}|z_{2}\rangle =1$ that leads to the separable state $|\psi_{3}\rangle =|z\rangle ⊗|z\rangle$. Furthermore, for C=1 (which characterizes entangled states), we have $P_{3}=\frac{1}{2}\;\Leftrightarrow \;\langle z_{1}|z_{2}\rangle =0$. Consequently, in the general case ($z_{1}$ and $z_{2}$ arbitrary), $P_{3}$ constitutes an alternative to the concurrence *C* for measuring the degree of entanglement of the general qutrit $|\psi_{3}\rangle$.

It is interesting to note that $P_{3}$ can be alternatively written as

$$P_{3}=\frac{1}{4}\left(3+n_{1}.n_{2}\right)$$

where the vectors

(60) $$n_{k}=\left(\frac{z_{k}+{\bar{z}}_{k}}{1+z_{k}{\bar{z}}_{k}},-i\frac{z_{k}-{\bar{z}}_{k}}{1+z_{k}{\bar{z}}_{k}},\frac{1-z_{k}{\bar{z}}_{k}}{1+z_{k}{\bar{z}}_{k}}\right)$$

(with k=1,2 and $i=\sqrt{-1}$) are unit vectors in the space $R^{3}$ which serve to locate points on the Bloch sphere. Therefore, entangled states are obtained for $n_{1}.n_{2}=-1$ (in this case, $P_{3}$ takes its minimal value $\frac{1}{2}$).

Note the following relation

(61) $$n_{i}.n_{j}=2{\left|\langle z_{i}|z_{j}\rangle \right|}^{2}-1$$

valid for arbitrary *i* and *j*. This relation will be useful for deriving closed-form expressions of $P_{d}$ in higher dimensional cases.

#### 5.3.3. Case d=4

In this case, the general state $|\psi_{4}\rangle$ of $G_{4}$ is made of N=3 qubits. It takes the form

$$\begin{array}{c} |\psi_{4}\rangle =c_{0}|3;0\rangle +c_{1}|3;1\rangle +c_{2}|3;2\rangle +c_{3}|3;3\rangle ,\;\sum_{k=0}^{3}{\left|c_{k}\right|}^{2}=1, \end{array}$$

where the Dicke states |3;k〉 with k=0,1,2,3 are

$$\begin{array}{ccc} |3;0\rangle & = & |0\rangle ⊗|0\rangle ⊗|0\rangle ,\\ |3;1\rangle & = & \frac{1}{\sqrt{3}}(|0\rangle ⊗|0\rangle ⊗|1\rangle +|0\rangle ⊗|1\rangle ⊗|0\rangle +|1\rangle ⊗|0\rangle ⊗|0\rangle ),\\ |3;2\rangle & = & \frac{1}{\sqrt{3}}(|0\rangle ⊗|1\rangle ⊗|1\rangle +|1\rangle ⊗|0\rangle ⊗|1\rangle +|1\rangle ⊗|1\rangle ⊗|0\rangle ),\\ |3;3\rangle & = & |1\rangle ⊗|1\rangle ⊗|1\rangle . \end{array}$$

In the Majorana representation, we have

$$\begin{array}{ccc} |N_{4}{|}^{-1}|\psi_{4}\rangle & = & |z_{1}\rangle ⊗|z_{2}\rangle ⊗|z_{3}\rangle +|z_{2}\rangle ⊗|z_{1}\rangle ⊗|z_{3}\rangle +|z_{1}\rangle ⊗|z_{3}\rangle ⊗|z_{2}\rangle \\ & + & |z_{3}\rangle ⊗|z_{2}\rangle ⊗|z_{1}\rangle +|z_{2}\rangle ⊗|z_{3}\rangle ⊗|z_{1}\rangle +|z_{3}\rangle ⊗|z_{1}\rangle ⊗|z_{2}\rangle , \end{array}$$

where the states $|z_{i}\rangle$ are given by Equation (41) with $z=z_{1},z_{2},z_{3}$ and the complex numbers $z_{1},z_{2},z_{3}$ are solutions of the polynomial equation of degree 3

$$\begin{array}{c} c_{0}z^{3}-\sqrt{3}c_{1}z^{2}+\sqrt{3}c_{2}z-c_{3}=0. \end{array}$$

The normalization factor $N_{4}$ reads

$$\begin{array}{c} |N_{4}{|}^{-2}=3!\mathrm{perm}\left(A_{3}\right)={\left(3!\right)}^{2}P_{4}, \end{array}$$

with

$$\begin{array}{c} P_{4}=\frac{1}{6}\left(1+|\langle z_{1}|z_{2}\rangle {|}^{2}+|\langle z_{2}|z_{3}\rangle {|}^{2}+{\left|\langle z_{3}|z_{1}\rangle \right|}^{2}+\langle z_{1}|z_{2}\rangle \langle z_{2}|z_{3}\rangle \langle z_{3}|z_{1}\rangle +\langle z_{1}|z_{3}\rangle \langle z_{3}|z_{2}\rangle \langle z_{2}|z_{1}\rangle \right) \end{array}$$

or alternatively

$$P_{4}=\frac{1}{6}\left(3+n_{1}.n_{2}+n_{2}.n_{3}+n_{3}.n_{1}\right),$$

where the components of the vectors $n_{i}$ (i=1,2,3) are given by Equation (60). From Equation (61), we get

$$P_{4}=\frac{1}{3}\left(\right|\langle z_{1}|z_{2}\rangle {|}^{2}+|\langle z_{2}|z_{3}\rangle {|}^{2}+|\langle z_{3}|z_{1}\rangle {|}^{2})$$

that clearly shows that

$$\begin{array}{c} \frac{1}{3}\leq P_{4}\leq 1 \end{array}$$

The case of complete separability corresponds to $P_{4}=1$. The minimal value $P_{4}=\frac{1}{3}$ is obtained for entangled states.

#### 5.3.4. Case d=5

In this case, the variables $z_{i}$ (i=1,2,3,4) are solutions of the equation of degree 4

$$c_{0}z^{4}-\sqrt{4}c_{1}z^{3}+\sqrt{6}c_{2}z^{2}-\sqrt{4}c_{3}z+c_{4}=0.$$

The calculation of $P_{5}$ yields

$$\begin{array}{ccc} P_{5} & = & \frac{1}{4!}(-6+4(|\langle z_{1}|z_{2}\rangle {|}^{2}+|\langle z_{1}|z_{3}\rangle {|}^{2}+|\langle z_{1}|z_{4}\rangle {|}^{2}+|\langle z_{2}|z_{3}\rangle {|}^{2}+|\langle z_{2}|z_{4}\rangle {|}^{2}+|\langle z_{3}|z_{4}\rangle {|}^{2})\\ & + & 2(|\langle z_{1}|z_{2}\rangle {|}^{2}|\langle z_{3}|z_{4}\rangle {|}^{2}+|\langle z_{1}|z_{3}\rangle {|}^{2}|\langle z_{2}|z_{4}\rangle {|}^{2}+|\langle z_{1}|z_{4}\rangle {|}^{2}|\langle z_{2}|z_{3}\rangle {|}^{2})) \end{array}$$

or

$$\begin{array}{ccc} P_{5} & = & \frac{1}{4!}(\frac{15}{2}+\frac{5}{2}\left(n_{1}.n_{2}+n_{1}.n_{3}+n_{1}.n_{4}+n_{2}.n_{3}+n_{2}.n_{4}+n_{3}.n_{4}\right)\\ & + & \frac{1}{2}\left(\left(n_{1}.n_{2}\right)\left(n_{3}.n_{4}\right)+\left(n_{1}.n_{3}\right)\left(n_{2}.n_{4}\right)+\left(n_{1}.n_{4}\right)\left(n_{2}.n_{3}\right)\right)) \end{array}$$

with

$$\frac{1}{4}\leq P_{5}\leq 1.$$

The minimal value $P_{5}=\frac{1}{4}$ can be obtained from

(62) $$\begin{array}{c} \langle z_{1}|z_{2}\rangle =\langle z_{1}|z_{3}\rangle =\langle z_{1}|z_{4}\rangle =0 \end{array}$$

or from any analogue equality deduced from Equation (62) by permutations of the indices 1,2,3,4.

#### 5.3.5. Case *d* Arbitrary

The general case is approached in Section 5.2. For *d* arbitrary, it can be shown that

$$\begin{array}{c} \frac{1}{N}\leq P_{d}\leq 1, \end{array}$$

the situation where $P_{d}=1$ corresponding to complete separability and $P_{d}=\frac{1}{N}$ to entangled states. Therefore, the parameter $P_{d}$ can serve as a measure of the entanglement of the symmetric qudit state $|\psi_{d}\rangle$ described by N=d−1 qubits.

The minimal value of $P_{d}$ can be obtained when

(63) $$\langle z_{1}|z_{2}\rangle =\langle z_{1}|z_{3}\rangle =\cdots =\langle z_{1}|z_{N}\rangle =0.$$

Thus, Equation (59) can be reduced to

$$P_{d}=\frac{1}{N!}\sum_{\sigma \in S_{N-1}}\prod_{i=2}^{N}\langle z_{i}|z_{\sigma (i)}\rangle .$$

The condition in Equation (63) implies that

$$z_{2}=z_{3}=\cdots =z_{N}.$$

In this case, we have

$$\sum_{\sigma \in S_{N-1}}\prod_{i=2}^{N}\langle z_{i}|z_{\sigma (i)}\rangle =\left(N-1\right)!$$

and the minimal value of $P_{d}$ is

$$P_{d}=\frac{(N-1)!}{N!}=\frac{1}{N}.$$

The same result can be obtained equally well, due to the invariance of $P_{d}$ under permutation symmetry, from any of the following conditions

$$\begin{array}{c} \langle z_{2}|z_{1}\rangle =\langle z_{2}|z_{3}\rangle =\cdots =\langle z_{2}|z_{N}\rangle =0,\\ \langle z_{3}|z_{1}\rangle =\langle z_{3}|z_{2}\rangle =\cdots =\langle z_{3}|z_{N}\rangle =0,\\ \vdots \\ \langle z_{N}|z_{1}\rangle =\langle z_{N}|z_{2}\rangle =\cdots =\langle z_{N}|z_{N-1}\rangle =0. \end{array}$$

instead of the condition in Equation (63).

## 6. Fubini–Study Metric

### 6.1. The Separable Case

The adequate approach to deal with the geometrical properties of a quantum state manifold is based on the derivation of the corresponding Fubini–Study metric [57]. The Fubini–Study metric is defined by the infinitesimal distance ds between two neighboring quantum states. This derivation is simplified by adopting the coherent states formalism. Indeed, for a single qubit coherent state |z〉 this is realized in the following way. Let us define the Kähler potential $K(\bar{z};z)$ as

(64) $$\begin{array}{c} K\left(\bar{z};z\right)=\ln{\left(\langle 0|z\rangle \right)}^{-2}. \end{array}$$

Using Equation (41) of the coherent state |z〉, we have

$$\begin{array}{c} K\left(\bar{z};z\right)=\ln\left(1+\bar{z}z\right) \end{array}$$

and the metric tensor

$$g=\frac{\partial^{2}K}{\partial z\partial \bar{z}}$$

becomes

$$g=\frac{1}{{\left(1+\bar{z}z\right)}^{2}},$$

so that the Fubini–Study metric $ds^{2}$ reads

$$ds^{2}=gdzd\bar{z}=\frac{1}{{\left(1+\bar{z}z\right)}^{2}}dzd\bar{z},$$

which coincides with the metric of the unit sphere. This provides us with a simple way to describe the 2-sphere $S^{2}$, or equivalently the complex projective space $CP^{1}$, usually regarded as the space of states of a $\frac{1}{2}$-spin particle.

This can be generalized to the completely separable state

$$|z_{1}z_{2}\cdots z_{N}\rangle =|z_{1}\rangle ⊗|z_{2}\rangle ⊗\cdots ⊗|z_{N}\rangle$$

constructed from the tensor product of *N* qubit coherent states. In this case, the Kähler potential is given by

(65) $$\begin{array}{c} K\left({\bar{z}}_{1}{\bar{z}}_{2}\cdots {\bar{z}}_{N};z_{1}z_{2}\cdots z_{N}\right)=\ln{\left(\langle 00\cdots 0|z_{1}z_{2}\cdots z_{N}\rangle \right)}^{-2}. \end{array}$$

This leads to

(66) $$\begin{array}{c} K\left({\bar{z}}_{1}{\bar{z}}_{2}\cdots {\bar{z}}_{N};z_{1}z_{2}\cdots z_{N}\right)=\sum_{i=1}^{N}\ln\left(1+{\bar{z}}_{i}z_{i}\right). \end{array}$$

The metric tensor *g* is defined via its components

$$g^{ij}=\frac{\partial^{2}K}{\partial z_{i}\partial {\bar{z}}_{j}}\;\Rightarrow \;g^{ij}=\delta_{i,j}\frac{1}{{\left(1+{\bar{z}}_{i}z_{i}\right)}^{2}}.$$

Finally, the Fubini–Study line element $ds^{2}$ is

(67) $$ds^{2}=g^{ij}dz_{i}d{\bar{z}}_{j}=\sum_{i=1}^{N}\frac{1}{{\left(1+{\bar{z}}_{i}z_{i}\right)}^{2}}dz_{i}d{\bar{z}}_{i}$$

associated with the complex space $CP^{1}\times CP^{1}\times \cdots \times CP^{1}$.

In the special case where the complex variables $z_{i}$ are identical, i.e., $z_{1}=z_{2}=\cdots =z_{N}=z$, the state $|z_{1}z_{2}\cdots z_{N}\rangle$ reduces to the coherent state given by Equation (42). In this case, the Fubini–Study metric takes the form

$$ds^{2}=N\frac{1}{{\left(1+\bar{z}z\right)}^{2}}dzd\bar{z},$$

which describes the unit 2-sphere, of radius $\sqrt{N}$, written in stereographic coordinates.

### 6.2. The Arbitrary Case

We now apply the just described geometrical picture to calculate the Fubini–Study metric for an arbitrary multiqubit symmetric state $|\psi_{d}\rangle$. Here, we define the Kähler potential through

$$\begin{array}{c} K\left({\bar{z}}_{1}{\bar{z}}_{2}\cdots {\bar{z}}_{N};z_{1}z_{2}\cdots z_{N}\right)=\ln{\left(\langle 00\cdots 0|\psi_{d}\rangle \right)}^{-2} \end{array}$$

as a generalization of Equations (64) and (65). It is easy to show that

$$\begin{array}{ccc} {\left(\langle 00\cdots 0|\psi_{d}\rangle \right)}^{-2} & = & \frac{1}{N!}\sum_{\sigma \in S_{N}}\prod_{i=1}^{N}\langle z_{i}|z_{\sigma (i)}\rangle \prod_{j=1}^{N}\left(1+{\bar{z}}_{j}z_{j}\right),\\ & = & \frac{1}{N!}\mathrm{perm}\left(A_{N}\right)\prod_{i=1}^{N}\left(1+{\bar{z}}_{i}z_{i}\right),\\ & = & P_{d}\prod_{i=1}^{N}\left(1+{\bar{z}}_{i}z_{i}\right). \end{array}$$

Hence, we obtain

$$\begin{array}{c} K\left({\bar{z}}_{1}{\bar{z}}_{2}\cdots {\bar{z}}_{N};z_{1}z_{2}\cdots z_{N}\right)=\ln P_{d}+\sum_{i=1}^{N}\ln\left(1+{\bar{z}}_{i}z_{i}\right) \end{array}$$

in terms of the parameter $P_{d}$ defined by Equation (59). As a result, the Kähler potential splits into two parts: one term is the Kähler potential corresponding to a completely separable state involving *N* qubits, cf. Equation (66), and the other term depends exclusively on the parameter $P_{d}$ which characterizes the degree of entanglement of the state $|\psi_{d}\rangle$. Then, the components of the corresponding metric tensor *g* are

$$\begin{array}{c} g^{ij}=\frac{\partial^{2}K}{\partial z_{i}\partial {\bar{z}}_{j}}=\frac{\partial^{2}\ln P_{d}}{\partial z_{i}\partial {\bar{z}}_{j}}+\delta_{i,j}\frac{1}{{\left(1+{\bar{z}}_{i}z_{i}\right)}^{2}} \end{array}$$

and the Fubini–Study line element $ds^{2}$ is

$$\begin{array}{c} ds^{2}=g^{ij}dz_{i}d{\bar{z}}_{j}=\frac{\partial^{2}\ln P_{d}}{\partial z_{i}\partial {\bar{z}}_{j}}dz_{i}d{\bar{z}}_{j}+\sum_{i=1}^{N}\frac{1}{{\left(1+{\bar{z}}_{i}z_{i}\right)}^{2}}dz_{i}d{\bar{z}}_{i}. \end{array}$$

In the special case where $P_{d}=1$, corresponding to a completely separable state, the last equation gives back Equation (67) valid for a multiqubit separable state. This is a further indication that the parameter $P_{d}$ encodes the geometrical aspects due to the entanglement of a multiqubit symmetric state.

## 7. Majorana Stars and Zeros of the Bargmann Function

### 7.1. The Main Idea

An arbitrary normalized state $|\psi_{d}\rangle$ of the space $G_{d}$ can be written either in terms of the Dicke states |N;k〉 with k=0,1,⋯,N=d−1 (see Equation (44)) or in terms of the coherent states $|z_{i}\rangle$ for i=1,2,⋯,N (see Equation (45)). The variables $z_{i}$, called Majorana stars [58], can be determined from the zeros of the Bargmann function ψ:z↦ψ(z) associated with the state $|\psi_{d}\rangle$. In fact, denoting by $\omega_{i}$ the zeros of the Bargmann function ψ, we shall show that the Majorana stars $z_{i}$ can be obtained from the Bargmann zeros $\omega_{i}$ via

$$\begin{array}{c} z_{i}=-\frac{1}{\omega_{i}},\;i=1,2,\cdots ,k_{\max}\leq N \end{array}$$

and we shall give the equation satisfied by the variables $z_{i}$.

### 7.2. Determining the Bargmann Zeros

In the analytic Fock–Bargmann representation [59], an arbitrary normalized state $|\psi_{d}\rangle$ of $G_{d}$ is represented by the Bargmann function ψ defined by

(68) $$\psi \left(z\right)=\langle N:\bar{z}|\psi_{d}\rangle ,$$

where the bra $\langle N:\bar{z}|$ follows from the coherent state

$$|N:z\rangle =\frac{1}{{\left(1+\bar{z}z\right)}^{\frac{N}{2}}}\sum_{k=0}^{N}z^{k}\sqrt{\frac{N!}{k!(N-k)!}}|N;k\rangle =|z\rangle ⊗|z\rangle ⊗\cdots ⊗|z\rangle$$

corresponding to the completely separable state in Equation (42). Thus, we have

$$\psi \left(z\right)=\frac{1}{{\left(1+\bar{z}z\right)}^{\frac{N}{2}}}\sum_{k=0}^{N}\sqrt{\frac{N!}{k!(N-k)!}}c_{k}z^{k},$$

which can be decomposed as

(69) $$\begin{array}{c} \psi \left(z\right)=\frac{1}{{\left(1+\bar{z}z\right)}^{\frac{N}{2}}}P\left(z\right), \end{array}$$

where

$$P\left(z\right)=\sum_{k=0}^{N}d_{k}z^{k},$$

with

$$d_{k}=\sqrt{\frac{N!}{k!(N-k)!}}c_{k}.$$

In fact, the polynomial

(70) $$\begin{array}{c} P\left(z\right)=\sum_{k=0}^{N}\sqrt{\frac{N!}{k!(N-k)!}}c_{k}z^{k} \end{array}$$

is of degree $k_{\max}\leq N$, where $k_{\max}$ is the maximum value of the index *k* for which $c_{k}\neq 0$. Therefore, the polynomial P(z) can be factorized as

$$P\left(z\right)=d_{k_{\max}}\left(z-\omega_{1}\right)\left(z-\omega_{2}\right)\cdots \left(z-\omega_{k_{\max}}\right)$$

where $\omega_{i}$ ($i=1,2,\cdots ,k_{\max}$) are called the Bargmann zeros.

### 7.3. Expression of $|\psi_{d}\rangle$ in Terms of the Bargmann Zeros

We now look for the expression of the state vector $|\psi_{d}\rangle$ in terms of the Bargmann zeros $\omega_{i}$ ($i=1,2,\cdots ,k_{\max}$). To this end, we remark that the scalar product between the state

$$|\omega_{i}\rangle =\frac{1}{\sqrt{1+{\bar{\omega }}_{i}\omega_{i}}}(|1\rangle -\omega_{i}\left|0\rangle \right)$$

and the coherent state $|\bar{z}\rangle \equiv |1:\bar{z}\rangle$ (see Equation (41)) is

$$\langle \bar{z}|\omega_{i}\rangle =\frac{z-\omega_{i}}{\sqrt{\left(1+\bar{z}z\right)\left(1+{\bar{\omega }}_{i}\omega_{i}\right)}},\;i=1,2,\cdots ,k_{\max}.$$

Thus, the polynomial P(z) can be written as

$$P\left(z\right)=d_{k_{\max}}{\left(1+\bar{z}z\right)}^{\frac{k_{\max}}{2}}\prod_{i=1}^{k_{\max}}\sqrt{1+{\bar{\omega }}_{i}\omega_{i}}\langle \bar{z}|\omega_{i}\rangle .$$

Furthermore, by noting that

$$\langle \bar{z}|0\rangle =\frac{1}{\sqrt{1+\bar{z}z}},$$

we extend the definition of the states $|\omega_{i}\rangle$ (initially defined for $i=1,2,\cdots ,k_{\max}$) by taking

$$|\omega_{i}\rangle =|0\rangle ,\;i=k_{\max}+1,k_{\max}+2,\cdots ,N,$$

so that the Bargmann function takes the form

$$\psi \left(z\right)=d_{k_{\max}}\prod_{i=1}^{k_{\max}}\sqrt{1+{\bar{\omega }}_{i}\omega_{i}}\prod_{j=1}^{N}\langle \bar{z}|\omega_{j}\rangle .$$

Since the representation $\psi_{d}\mapsto \psi$ is unique up to permutations of the $|\omega_{i}\rangle$, the Bargmann function can be rewritten as

$$\psi \left(z\right)=d_{k_{\max}}\prod_{i=1}^{k_{\max}}\sqrt{1+{\bar{\omega }}_{i}\omega_{i}}\frac{1}{N!}\sum_{\sigma \in S_{N}}\prod_{j=1}^{N}\langle \bar{z}|\omega_{\sigma (j)}\rangle$$

or alternatively as

(71) $$\begin{array}{c} \psi \left(z\right)=N_{d}\sum_{\sigma \in S_{N}}\langle N:\bar{z}|\omega_{\sigma (1)}\omega_{\sigma (2)}\cdots \omega_{\sigma (N)}\rangle , \end{array}$$

where the normalization constant $N_{d}$ is given by

$$N_{d}=\frac{1}{N!}d_{k_{\max}}\prod_{i=1}^{k_{\max}}\sqrt{1+{\bar{\omega }}_{i}\omega_{i}}.$$

Comparing Equations (68) and (71), we find that the state $|\psi_{d}\rangle$ can be expressed as

(72) $$\begin{array}{c} |\psi_{d}\rangle =N_{d}\sum_{\sigma \in S_{N}}\sigma (|\omega_{1}\rangle ⊗|\omega_{2}\rangle ⊗\cdots ⊗|\omega_{N}\rangle ) \end{array}$$

in terms of the $k_{\max}$ zeros $\omega_{i}$ for $i=1,2,\cdots ,k_{\max}$ of the Bargmann function ψ and of their extension $\omega_{k_{\max+1}}=\omega_{k_{\max+2}}=\cdots =\omega_{N}=0$.

### 7.4. Expression of $|\psi_{d}\rangle$ in Terms of the Majorana Stars

We note that the states $|\omega_{i}\rangle$ with $i=1,2,\cdots ,k_{\max}$ can be written in terms of the coherent states $|z_{i}\rangle \equiv |1:z_{i}\rangle$ by putting

$$\begin{array}{c} z_{i}=\left\{\begin{array}{c} -\frac{1}{\omega_{i}}\;\mathrm{if}\;i=1,2,\cdots ,k_{\max},\\ 0\;\mathrm{if}\;i=k_{\max}+1,k_{\max}+2,\cdots ,N. \end{array}\right. \end{array}$$

We verify that

$$|\omega_{i}\rangle =|z_{i}\rangle ,\;i=1,2,\cdots ,N$$

up to irrelevant phase factors. Hence, the symmetric qudit state $|\psi_{d}\rangle$ given by Equation (72) can be expressed as

(73) $$\begin{array}{c} |\psi_{d}\rangle =N_{d}\sum_{\sigma \in S_{N}}\sigma (|z_{1}\rangle ⊗|z_{2}\rangle ⊗\cdots ⊗|z_{N}\rangle ) \end{array}$$

in terms of the coherent states $|z_{i}\rangle$. Equation (73) is identical to Equation (45); thus, we recover Equation (45).

### 7.5. Equation Satisfied by the Majorana Stars

The zeros $\omega_{i}$ of the Bargmann function in Equation (69) satisfy $P(\omega_{i})=0$. From Equation (70), we thus get

$$\begin{array}{c} \sum_{k=0}^{k_{\max}}\sqrt{\frac{N!}{k!(N-k)!}}c_{k}\omega_{i}^{k}=0\;\Rightarrow \;\sum_{k=0}^{N}\sqrt{\frac{N!}{k!(N-k)!}}c_{k}\omega_{i}^{k}=0 \end{array}$$

or in terms of the $z_{i}$

$$\sum_{k=0}^{k_{\max}}{\left(-1\right)}^{k}\sqrt{\frac{N!}{k!(N-k)!}}c_{k}z_{i}^{N-k}=0\;\Rightarrow \;\sum_{k=0}^{N}{\left(-1\right)}^{k}\sqrt{\frac{N!}{k!(N-k)!}}c_{k}z_{i}^{N-k}=0$$

in agreement with Equation (56).

## 8. Concluding Remarks

In this study, we discussed the role of a specific generalized Weyl–Heisenberg algebra in the algebraic structure of qubits and qudits. The use of this generalized Weyl–Heisenberg algebra was based on the fact that qubits are neither fermions nor bosons. Indeed, in the standard theoretical approach of quantum information, a qubit is a vector in a two-dimensional Hilbert space as for fermions and the Hilbert space of a multiqubit system has a tensor product structure similar to for bosons. In this respect, the commutation rules of the raising and lowering operators for qubits are not specified by relations of bosonic type or of fermionic type.

By using a collection of N=d−1 qubits, we gave a realization of the *d*-dimensional representation space of the generalized Weyl–Heisenbeg algebra. In particular, we demonstrated that the vectors of this representation space coincide with the Dicke states. These states are of special interest for describing multiqubit quantum systems possessing exchange symmetry. Another advantage of this algebraic description via the generalized Weyl–Heisenberg algebra concerns the separability of multiqubit states invariant under permutations. Hence, starting from the decomposition of Dicke states, we investigated the condition for the separability of symmetric qudits made of N=d−1 qubit states. Our results show that exchange symmetry implies that the superposition of Dicke states are globally entangled unless they are fully separable and coincide with the coherent states, in the Perelomov sense, associated with the generalized Weyl–Heisenberg algebra.

In the Majorana description of a symmetric qudit state in terms of symmetrized tensor products of N=d−1 qubits, we introduced a parameter $P_{d}$ connected to the permanent of the matrix characterizing the overlap between the *N* qubits. This parameter provides us with a quantitative measure of the entanglement for the qudit arising from *N* qubits. This was illustrated in the special case d=3, for which the parameter $P_{d}$ constitutes an alternative to the Wootters concurrence *C* for N=2 qubits. Therefore, we propose that $P_{d}$ be called *perma-concurrence* as a contraction of permanent and concurrence. Other examples of $P_{d}$ were given for d=4 and 5. The results highlight the interest of the perma-concurrence $P_{d}$ for measuring the entanglement of a symmetric qudit state developed in terms of tensor products of qubit coherent states.

In Section 7, we further investigated the formalism of qubit coherent states to describe qudit states in the Fock–Hilbert space corresponding to the generalized Weyl–Heisenberg algebra. More precisely, we used the Fock–Bargmann representation for describing any symmetric qudit constructed from N=d−1 qubits with the help of an analytic function, the so-called Bargmann function. The zeros of the Bargmann function were related to the Majorana stars which provide an alternative way to describe Fock–Hilbert states as tensor products of qubit coherent states labeled by complex variables, namely, Majorana stars on the Bloch sphere.

Recently, new entropic and information inequalities for one qudit, which differs from a multiqubit system, have been developed [60]. Therefore, it will be a challenge to ask whether the qudit picture proposed in this paper can be adapted in terms of linear combinations of Dicke states.

To close this paper, note that it might be interesting to introduce Dicke states into the construction of the so-called mutually unbiased bases used in quantum information. This approach, feasible in view of the connection between mutually unbiased bases and angular momentum states [61], could be the object of a future work.

## References

[1] Kimura G. (2003). The Bloch vector for *N*-level systems. Phys. Lett. A.

[2] Kimura G., Kossakowski A. (2005). The Bloch-vector space for *N*-level systems—The spherical-coordinate point of view. Open Syst. Inf. Dyn..

[3] Bertlmann R.A., Krammer P. (2008). Bloch vectors for qudits. J. Phys. A Math. Theor..

[4] Majorana E. (1932). Atomi orientati in campo magnetico variabile. Nuovo Cimento.

[5] Dür W., Vidal G., Cirac J.I. (2000). Three qubits can be entangled in two inequivalent ways. Phys. Rev. A.

[6] Verstraete F., Dehaene J., De Moor B., Verschelde H. (2002). Four qubits can be entangled in nine different ways. Phys. Rev. A.

[7] Bastin T., Krins S., Mathonet P., Godefroid M., Lamata L., Solano E. (2009). Operational families of entanglement classes for symmetric *N*-qubit states. Phys. Rev. Lett..

[8] Ekert A.K. (1991). Quantum cryptography based on Bell’s theorem. Phys. Rev. Lett..

[9] Bennett C.H., Brassard G., Crépeau C., Jozsa R., Peres A., Wootters W.K. (1993). Teleporting an unknown quantum state via dual classical and Einstein-Podolsky-Rosen channels. Phys. Rev. Lett..

[10] Keyl M., Werner R.F., Buchleitner A., Hornberger K. (2002). How to correct small quantum errors. Coherent Evolution in Noisy Environments.

[11] Weedbrook C., Pirandola S., García-Patrón R., Cerf N.J., Ralph T.C., Shapiro J.H., Lloyd S. (2012). Gaussian quantum information. Rev. Mod. Phys..

[12] Hill S., Wootters W.K. (1997). Entanglement of a pair of quantum bits. Phys. Rev. Lett..

[13] Wootters W.K. (1998). Entanglement of formation of an arbitrary state of two qubits. Phys. Rev. Lett..

[14] Coffman V., Kundu J., Wootters W.K. (2000). Distributed entanglement. Phys. Rev. A.

[15] Ganczarek W., Kuś M., Życzkowski K. (2012). Barycentric measure of quantum entanglement. Phys. Rev. A.

[16] Anandan J. (1991). A geometric approach to quantum mechanics. Found. Phys..

[17] Schilling T. (1996). Geometry of Quantum Mechanics. Ph.D. Thesis.

[18] Ashtekar A., Schilling T.A., Harvey A. (1999). Geometrical formulation of quantum mechanics. On Einstein’s Path.

[19] Brody D.C., Hughston L.P. (2000). Geometric quantum mechanics. J. Geom. Phys..

[20] Kuś M., Życzkowski K. (2001). Geometry of entangled states. Phys. Rev. A.

[21] Bengtsson I., Brännlund J., Życzkowski K. (2002). ℂ*P^n^*, or, entanglement illustrated. Int. J. Mod. Phys. A.

[22] Brody D.C., Gustavsson A.C.T., Hughston L.P. (2007). Entanglement of three-qubit geometry. J. Phys. Conf. Ser..

[23] Sakajii A., Licata I., Singh J., Felloni S. (2010). New Trends in Quantum Information.

[24] Mosseri R., Dandoloff R. (2001). Geometry of entangled states, Bloch spheres and Hopf fibrations. J. Phys. A Math. Gen..

[25] Wei T., Goldbart P.M. (2003). Geometric measure of entanglement and applications to bipartite and multipartite quantum states. Phys. Rev. A.

[26] Hubener R., Kleinmann M., Wei T.-C., González-Guillén C., Gühne O. (2009). Geometric measure of entanglement for symmetric states. Phys. Rev. A.

[27] Aulbach M., Markham D., Murao M. (2010). The maximally entangled symmetric state in terms of the geometric measure. New J. Phys..

[28] Martin J., Giraud O., Braun P.A., Braun D., Bastin T. (2010). Multiqubit symmetric states with high geometric entanglement. Phys. Rev. A.

[29] Chen L., Aulbach M., Hajdušek M. (2014). Comparison of different definitions of the geometric measure of entanglement. Phys. Rev. A.

[30] Baguette D., Bastin T., Martin J. (2014). Multiqubit symmetric states with maximally mixed one-qubit reductions. Phys. Rev. A.

[31] Miyake A. (2003). Classification of multipartite entangled states by multidimensional determinants. Phys. Rev. A.

[32] Heydari H. (2008). Geometrical structure of entangled states and the secant variety. Quantum Inf. Process..

[33] Holweck F., Luque J.-G., Thibon J.-Y. (2012). Geometric descriptions of entangled states by auxiliary varieties. J. Math. Phys..

[34] Radcliffe J.M. (1971). Some properties of coherent spin states. J. Phys. A Gen. Phys..

[35] Mandilara A., Coudreau T., Keller A., Milman P. (2014). Entanglement classification of pure symmetric states via spin coherent states. Phys. Rev. A.

[36] Stockton J.K., Geremia J.M., Doherty A.C., Mabuchi H. (2003). Characterizing the entanglement of symmetric many-particle spin-$\frac{1}{2}$ systems. Phys. Rev. A.

[37] Mathonet P., Krins S., Godefroid M., Lamata L., Solano E., Bastin T. (2010). Entanglement equivalence of *N*-qubit symmetric states. Phys. Rev. A.

[38] Markham D.J.H. (2011). Entanglement and symmetry in permutation-symmetric states. Phys. Rev. A.

[39] Augusiak R., Tura J., Samsonowicz J., Lewenstein M. (2012). Entangled symmetric states of *N* qubits with all positive partial transpositions. Phys. Rev. A.

[40] Aulbach M. (2012). Classification of entanglement in symmetric states. Int. J. Quantum Inf..

[41] Novo L., Moroder T., Gühne O. (2013). Genuine multiparticle entanglement of permutationally invariant states. Phys. Rev. A.

[42] Tóth G., Wieczorek W., Gross D., Krischek R., Schwemmer C., Weinfurter H. (2010). Permutationally invariant quantum tomography. Phys. Rev. Lett..

[43] Moroder T., Hyllus P., Tóth G., Schwemmer C., Niggebaum A., Gaile S., Gühne O., Weinfurter H. (2012). Permutationally invariant state reconstruction. New J. Phys..

[44] Klimov A.B., Björk G., Sánchez-Soto L.L. (2013). Optimal quantum tomography of permutationally invariant qubits. Phys. Rev. A.

[45] Dicke R. (1954). Coherence in spontaneous radiation processes. Phys. Rev..

[46] Tóth G. (2007). Detection of multipartite entanglement in the vicinity of symmetric Dicke states. J. Opt. Soc. Am. B.

[47] Bergmann M., Gühne O. (2013). Entanglement criteria for Dicke states. J. Phys. A Math. Theor..

[48] Daoud M., Kibler M.R. (2006). Fractional supersymmetry and hierarchy of shape invariant potentials. J. Math. Phys..

[49] Daoud M., Kibler M.R. (2010). Phase operators, temporally stable phase states, mutually unbiased bases and exactly solvable quantum systems. J. Phys. A Math. Theor..

[50] Daoud M., Kibler M.R. (2011). Phase operators, phase states and vector phase states for *SU*_3_ and *SU*_2,1_. J. Math. Phys..

[51] Wu L.-A., Lidar D.A. (2002). Qubits as parafermions. J. Math. Phys..

[52] Frydryszak A.M. (2008). Nilpotent quantum mechanics, qubits, and flavors of entanglement. arXiv.

[53] Palev T.D. (1977). Lie Algebraical Aspects of Quantum Statistics. Unitary Quantization (A-Quantization).

[54] Green H.S. (1953). A generalized method of field quantization. Phys. Rev..

[55] Condon E.U., Odabaşi H. (1980). Atomic Structure.

[56] Daoud M., Kibler M.R. (2012). Bosonic and k-fermionic coherent states for a class of polynomial Weyl-Heisenberg algebras. J. Phys. A Math. Theor..

[57] Anandan J., Aharonov Y. (1990). Geometry of quantum evolution. Phys. Rev. Lett..

[58] Bacry H. (1980). Constellations and projective classical groups. Commun. Math. Phys..

[59] Bargmann V. (1961). On a Hilbert space of analytic functions and an associated integral transform. Commun. Pure Appl. Math..

[60] Man’ko M.A., Man’ko V.I. (2014). The quantum strong subadditivity condition for systems without subsystems. Phys. Scr..

[61] Kibler M.R., Sakajii A., Licata I., Singh J., Felloni S. (2010). Formulas for mutually unbiased bases in systems of qudits. New Trends in Quantum Information.

